# Coenzyme biosynthesis in response to precursor availability reveals incorporation of β-alanine from pantothenate in prototrophic bacteria

**DOI:** 10.1016/j.jbc.2023.104919

**Published:** 2023-06-12

**Authors:** Birgitta Ryback, Julia A. Vorholt

**Affiliations:** Institute of Microbiology, ETH Zurich, Zurich, Switzerland

**Keywords:** bacterial metabolism, coenzyme A, β-alanine, nicotinamide adenine dinucleotide (NAD), NAD biosynthesis, vitamin, metabolic tracer, metabolomics, microbiome, mass spectrometry (MS), flavin, pyridoxal phosphate, coenzyme metabolism, overflow metabolism, salvage pathways

## Abstract

Coenzymes are important for all classes of enzymatic reactions and essential for cellular metabolism. Most coenzymes are synthesized from dedicated precursors, also referred to as vitamins, which prototrophic bacteria can either produce themselves from simpler substrates or take up from the environment. The extent to which prototrophs use supplied vitamins and whether externally available vitamins affect the size of intracellular coenzyme pools and control endogenous vitamin synthesis is currently largely unknown. Here, we studied coenzyme pool sizes and vitamin incorporation into coenzymes during growth on different carbon sources and vitamin supplementation regimes using metabolomics approaches. We found that the model bacterium *Escherichia coli* incorporated pyridoxal, niacin, and pantothenate into pyridoxal 5′-phosphate, NAD, and coenzyme A (CoA), respectively. In contrast, riboflavin was not taken up and was produced exclusively endogenously. Coenzyme pools were mostly homeostatic and not affected by externally supplied precursors. Remarkably, we found that pantothenate is not incorporated into CoA as such but is first degraded to pantoate and β-alanine and then rebuilt. This pattern was conserved in various bacterial isolates, suggesting a preference for β-alanine over pantothenate utilization in CoA synthesis. Finally, we found that the endogenous synthesis of coenzyme precursors remains active when vitamins are supplied, which is consistent with described expression data of genes for enzymes involved in coenzyme biosynthesis under these conditions. Continued production of endogenous coenzymes may ensure rapid synthesis of the mature coenzyme under changing environmental conditions, protect against coenzyme limitation, and explain vitamin availability in naturally oligotrophic environments.

Coenzymes are essential metabolites that serve as catalytically active units. They expand the catalytic repertoire of enzymes and are concomitantly required by around half of the known enzymes (1) Recently, it was demonstrated that the carbon backbones of coenzymes are stable *in vivo* and cells only need to synthesize them to compensate for dilution by growth (2). To meet this extraordinary longevity, coenzyme biosynthesis is tightly regulated. Known molecular mechanisms to control coenzyme biosynthesis include riboswitches (thiamine, flavin cofactors, cobalamine cofactors), transcriptional regulation (NAD, biotin), allosteric inhibition (PLP), or competitive enzyme inhibition (CoA) (3, 4, 5, 6, 7, 8).

Many coenzymes are produced from dedicated precursors, which are either provided in the environment or are synthesized from simple carbon and nitrogen sources (1). Cells synthesize some of their coenzymes from soluble precursors, referred to as the B group vitamins. These vitamins include thiamine, riboflavin, niacin, pantothenate, pyridoxal, biotin, folate, and cobalamin (9). In recent years, biotechnological processes have been set up for the production of many of these vitamins (10). Vitamin overproduction strains are readily obtained by overexpressing genes encoding the appropriate enzymes, whereas coenzyme overproduction requires overcoming regulatory mechanisms (10, 11, 12), which implies that regulation of coenzyme biosynthesis generally targets reactions converting the vitamin precursor to the mature coenzyme. Bacteria are also able to regulate their metabolism based on nutrient availability: mechanisms have been described for amino acids, carbon sources, and FAD (a mature coenzyme) but so far not for coenzyme precursors (13).

Cells keep most metabolite pools constant between growth conditions, indicating homeostasis of metabolite concentrations (14, 15, 16, 17). Intracellular concentrations of individual coenzymes such as NAD(P) or CoA have a wide range, NAD being the highest at about 1 mM (18) and PLP being present at lower concentration of 100 μM (16). The flavin cofactors FAD and FMN are estimated to be present at 100 μM and 10 μM, respectively (18). CoA (free and acetylated) concentrations have been reported to be in the range of 1 to 10 mM (18, 19). Whether the physiological state of the cell determines coenzyme pools is not well established. A comparison of coenzyme pools between carbon sources suggests that NAD(P), FAD, and FMN vary little when cells were grown on glucose, glycerol, and acetate (18). The total CoA pool is stable, although the dominant acyl-CoA species varies depending on the carbon source (19). Effects of vitamin supplementation on pools of intracellular coenzymes have not been systematically studied, but the NAD(P) pool increases up to twofold when *Escherichia coli* cultures are supplemented with niacin (2). This finding raises the question of the extent to which coenzyme pools are affected by the carbon source, precursor availability in the medium, or the interplay between the two.

Many bacteria are auxotrophic for vitamins, meaning that they are unable to synthesize coenzyme precursors and rely on external vitamin supply (20, 21, 22, 23, 24, 25, 26, 27). However, also prototrophic bacteria can potentially use exogenous coenzyme precursors if they are provided in the environment. It is currently unknown to which extent vitamin supplements are taken up and incorporated into coenzymes and whether bacteria systematically downregulate vitamin biosynthesis in the presence of externally supplemented vitamins. In the present study, we address the following questions: (i) Do pool sizes of coenzymes change as a function of external vitamin supply? (ii) Do prototrophic bacteria use externally supplied vitamins to produce coenzymes? and if so (iii) Do bacteria continue to produce coenzyme precursors in the presence of external vitamin supply? To investigate these questions we use the laboratory strain of *E. coli* as a model and apply LC/MS-based metabolomics in combination with metabolic tracer analysis (28) and isotope dilution mass spectrometry (29). We find that coenzyme concentrations are largely homeostatic. External vitamins are incorporated for most of the coenzymes tested. We also demonstrate concentration of vitamins at which the intracellular coenzyme pools are fully derived from external vitamins. We then reveal that vitamins are produced in excess, as measured by secretion of vitamins to the growth media. In addition, we uncover that *E. coli* preferentially utilizes the β-alanine moiety of pantothenic acid instead of intact pantothenate to generate CoA. We then show that this preference for β-alanine also occurs in additional prototrophic environmental strains and propose a conserved mechanism for partial scavenging of pantothenic acid.

## Results

### Effect of growth medium on coenzyme pools

**Figure 1:**
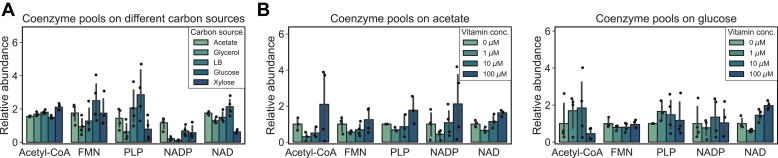
**Coenzyme pool sizes in *E. coli* under varying conditions.***A*, coenzyme pools of cells grown on different carbon sources, which were selected to range from 0.3 h^−1^ (acetate) to 2.1 h^−1^ (LB). *B*, coenzyme pools of cells upon growth in the presence of externally provided vitamins during growth on acetate and glucose, respectively. Quantification is provided relative to U^13^C-labeled *E. coli* extract.

Coenzymes or their precursors can be stored in cells, making it possible for cells to increase their internal reservoir of these essential metabolites in response to environmental stimuli (2, 27). Here, we addressed whether coenzyme pool sizes change in response to external vitamin concentration or carbon substrate using *E. coli* as the model organism. We observed that coenzyme pools were similar in different carbon sources, indicating that coenzyme pools are not dependent on physiology or growth rate ranging from 0.3 h^−1^ to 2.1 h^−1^ (30, 31) (Fig. 1*A*), which confirms earlier reports in which coenzyme pools were found relatively conserved between conditions (16, 18, 19).

We then tested the effect of vitamin supplementation on coenzyme pool sizes by acquiring metabolomics data from cells that were grown in the presence of different externally supplied vitamin concentrations under standardized growth conditions using glucose as a carbon source. To probe for the potential interplay between vitamin supplementation and physiology, we repeated the experiment with acetate as the carbon source. The growth rate of *E. coli* was generally robust to changes in vitamin supplementation except for growth on glucose, where the growth rate decreased by 20% when vitamin concentration was increased from 10 to 100 μM (Fig. S1). For CoA, flavin coenzymes, and PLP, we observed that vitamin supplementation did not affect the intracellular pool size of the corresponding coenzyme (Fig. 1*B*). We found that the concentration of NAD(H) increased up to twofold (Fig. 1*B*), consistent with earlier observations (2). These results were comparable when cells were grown on glucose and acetate and indicate that NAD(H) pools are increased in response to environmentally added niacin.

Fluxes through enzyme-catalyzed reactions are sensitive to the concentration of their reactants. In particular, metabolite concentrations can be used to infer reaction kinetics *in vivo*, because reactions for which the substrate or product concentration is close to the K_m_ of the enzyme are sensitive to changes in pool sizes (14). Since we observed that NAD(H) was the only coenzyme whose concentration increased in response to precursor supplementation, we investigated whether increased NAD(H) concentration could lead to saturation of more NAD(H)-dependent reactions by retrieving K_m_ data from the BRENDA database (32). We then projected the K_m_ values against two scenarios: one in which NAD(H) concentration was as calculated before (18) and one in which these values were increased by factor of 2 as was observed between the lowest and highest NAD(H) measurement in Figure 1*B*. We found that a few enzymes in central metabolism would be saturated upon the increase in NAD(H) (Table S1), potentially increasing the rate of these reactions *in vivo*.

### *E. coli* uses supplemented precursors of NAD and PLP

**Figure 2:**
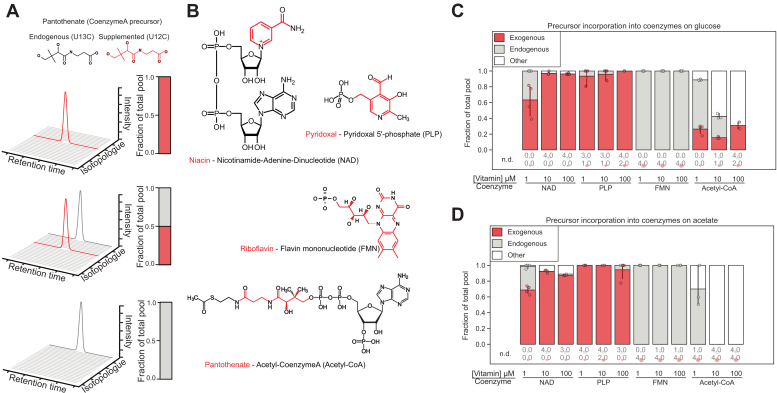
**Vitamin incorporation into *E. coli*.***A*, a schematic of data interpretation. In the presence of supplemented vitamins and U^13^C carbon source, cells can incorporate the endogenous (and hence heavy) vitamin or the lighter supplement. Coenzymes produced from either of the two vitamin isotopologues can be distinguished using mass spectrometry. For each of the *N* + 1 possible isotopologues, the fraction of the total pool is calculated as the peak area of that isotopologue divided by the sum of peak areas of all *N* + 1 isotopologues. These fractions are presented in stacked barplots. In this example trace, there are only two major traces contributing to the total pool of the compound, and the total abundance of the remaining *N*-2 isotopologues is 0. *B*, structures of the four vitamin-coenzyme pairs included in this study. The vitamin moiety is highlighted in *red*. *C*, the contribution of a vitamin supplement and endogenous vitamin moiety to the total pool of each coenzyme in cultures using ^13^C glucose as a carbon source. The area under the vitamin-derived and fully endogenous traces (such as in *A*) are divided by the total pool (sum under all *N* + 1 isotopologue traces). “Other” refers to traces that do not correspond to the *m/z* of either of the two expected dominant traces. *D*, contribution of vitamin supplement to the total coenzyme pool when ^13^C acetate was used as C source.

When vitamins are available in the environment, prototrophs may take them up, continue to produce them endogenously, or both. To distinguish between these scenarios, we grew *E. coli* in liquid cultures supplied with ^13^C glucose and supplemented the cultures either with 1, 10, or 100 μM naturally labeled (^12^C) precursors for more than ten doublings to reach steady state conditions. We extracted the intracellular metabolites from exponentially growing cells and measured the label distribution in the corresponding coenzymes. A mass shift thus allows us to distinguish between coenzymes that were synthesized entirely endogenously or from the external vitamin source. Observing fully labeled coenzymes indicates *de novo* biosynthesis from central metabolites, while coenzymes with unlabeled carbon atoms indicate the incorporation of external vitamins (Fig. 2*A*). We focused on four vitamin–coenzyme pairs (Fig. 2*B*). When vitamins were supplemented at 1 μM concentration, we found that NAD and PLP were predominantly produced from external vitamin precursor sources, and at 10 and 100 μM these two coenzymes were entirely generated from the supplements (Fig. 2*C*). The labeling pattern of FAD or FMN indicated that riboflavin was not incorporated at any of the concentrations tested (Figs. 2*C* and S2). With 1 μM pantothenate supplementation, 20% of the intracellular (acetyl-)CoA pool was derived from the supplement, 70% from endogenous pantothenate, and 10% from an isotopologue other than the former two. With increasing pantothenate concentration, the U^13^C-labeled endogenous fraction of CoA decreased while the pantothenate-derived fraction remained at 15 to 20% of the total pool (labeled in gray and red in Fig. 2*C*). With 10 μM or 100 μM pantothenate, more than half of the CoA pool corresponded to an *m/z* trace other than the two expected isotopologues.

To investigate the interplay between physiology and coenzyme biosynthesis, we studied vitamin incorporation into coenzymes in acetate-grown cells which divide at a slower rate. We found that niacin, pyridoxal, and riboflavin incorporation was comparable to cells grown in glucose (Fig. 2*D*). For pantothenate incorporation, we observed that even with the lowest pantothenate concentration, the majority of (acetyl-)CoA pool was neither synthesized from endogenous pantothenate nor was it the product of vitamin incorporation (Fig. 2*D*). With 10 and 100 μM pantothenate, 100% of intracellular CoA pool was found labeled with an unexpected isotopologue. Because pantothenate incorporation to CoA is different when cells are grown on glucose and acetate, supplement incorporation depends on growth rate and/or physiology, and proceeds *via* a previously uncharacterized precursor. The same pattern was observed in free CoA and succinyl-CoA (Fig. S3).

### β-alanine moiety of pantothenate is incorporated into coenzyme A

**Figure 3:**
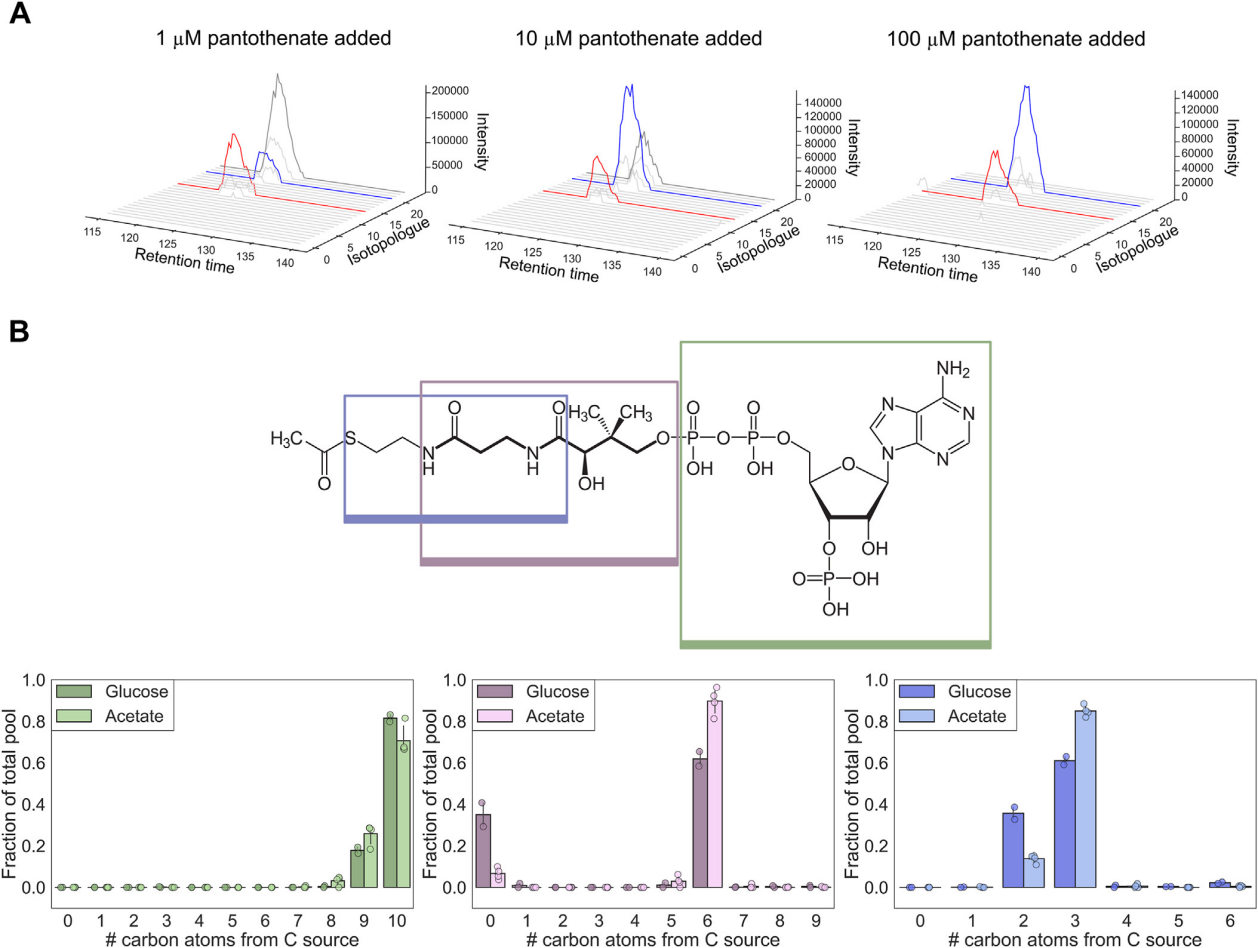
**β-alanine incorporated into CoA from pantothenate.***A*, extracted ion chromatograms of all possible acetyl-CoA isotopologues in cells grown on ^13^C glucose in the presence of 1, 10, and 100 μM pantothenate. *B*, acetyl-CoA molecules were fragmented and label incorporation in three representative fragments was studied. Fragments are depicted in *rectangles* overlaid on the acetyl-CoA structure. Pantothenate moiety of the CoA backbone is depicted in *boldface*. Adenosine fragment (10C; shown in *green*) was expected to be U^13^C labeled as it includes no carbon atoms from the pantothenate supplement. Pantothenate fragment (9C; *purple*) was expected to be U^13^C labelled if the supplement was not incorporated, U^12^C labelled (0 × ^13^C) if supplement was incorporated, and 6 × ^13^C labeled if there were three ^12^C atoms incorporated from the supplement. The last fragment includes the β-alanine moiety of pantothenate. This fragment shows whether the three ^12^C atoms in the pantothenate moiety originate from β-alanine (in which case this fragment would be 0 × ^13^C labeled) or pantoate (in which case this fragment would be 5 × ^13^C labeled).

We observed that the intracellular acetyl-CoA pool extracted from *E. coli* cells grown on ^13^C carbon source and ^12^C pantothenate was derived neither from supplemented pantothenate nor from endogenous synthesis, which prompted us to further investigate the origin of carbon atoms within the CoA molecule. Acetyl-CoA was further analyzed as it was the most abundant CoA species. Closer inspection of all 24 acetyl-CoA isotopologues (0 × ^13^C to 23 × ^13^C) in samples from cultures grown on ^13^C-glucose supplemented with 1 μM ^12^C-pantothenate revealed three major isotopologues contributing to the CoA pool. Two of them were the expected isotopologues: fully-labeled (*M*) and *M*-9 for CoA incorporating endogenous and supplement-derived pantothenate, respectively (red and gray traces in Fig. 3*A*). The third trace corresponded to the *m*/*z* of isotopologue *M*-3 (blue in Fig. 3*A*), which indicated the incorporation of three ^12^C units into the mature coenzyme.

Having determined the number of ^12^C units incorporated into CoA from vitamin supplements, we next wanted to confirm that the three naturally labeled carbon atoms were indeed derived from pantothenate, and determine which part of the vitamin molecule was incorporated into the coenzyme. Because pantothenate (9C) consists of two moieties, pantoate (6C) and β-alanine (3C) (33, 34), we addressed whether the three ^12^C atoms originated from the β-alanine moiety. To answer this question, we employed an MS^2^ approach, whereby all expected acetyl-CoA isotopologues were fragmented and subsequently the *m/z* values of these fragments were monitored. Within these data, we identified three informative fragments (Fig. 3*B* overlay over the acetyl-CoA structure). The first fragment corresponded to the expected *m/z* of the acyl-pantetheine fragment, and it contained three ^12^C atoms (Fig. 3*B*; purple). The second fragment contained the adenosine moiety of the coenzyme, which was expected and confirmed to be fully derived from endogenous metabolism (Fig. 3*B*; green). Together, the two fragments confirmed that these three carbon atoms are derived from the acyl-pantetheine moiety. The third fragment corresponded to a substructure of acyl-pantetheine that contains the intact β-alanine moiety. This fragment was also labeled with three ^12^C units, suggesting that the unexpected CoA isotopologue contained ^12^C from β-alanine (Fig. 3*B*; blue). Because the only source of ^12^C β-alanine was the externally provided pantothenate supplement, we hypothesize that pantothenate is cleaved, which leads to free ^12^C-β-alanine and subsequent condensation with endogenous ^13^C-pantoate into a chimeric pantothenate molecule.

### Vitamin biosynthesis upon vitamin supplementation

**Figure 4:**
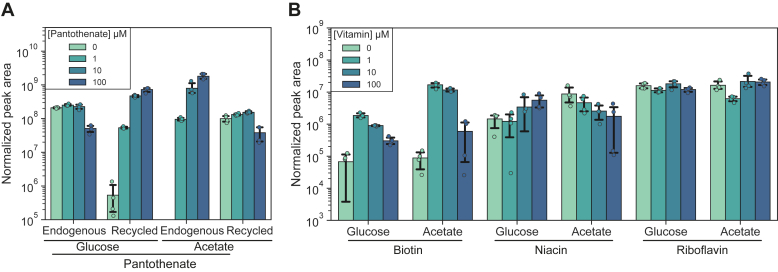
**Vitamin secretion in the presence of vitamin supplements.** Vitamin supplements (U^12^C) were present at concentrations indicated in the legend. *A*, presence of pantothenate in spent media. Baseline normalized peak areas are shown for two isotopologues: endogenous (9 × ^13^C, [M-H]^−^*m/z* 227.1337) pantothenate and recycled pantothenate (6 × ^13^C + 3 × ^12^C, [M-H]^−^*m/z* 224.1236). *B*, biotin, niacin, and riboflavin. For each vitamin, baseline normalized peak areas are shown for endogenous (U^13^C) vitamin.

While monitoring ^12^C vitamin incorporation into otherwise ^13^C-labeled coenzymes, we observed that only the β-alanine moiety of supplemented pantothenate was incorporated into CoA (Figs. 2 and 3). We next wanted to address whether this pattern was also present in the free pantothenate pool. As pantothenate is secreted to the growth medium due to the overflow mechanism (6, 35), we studied the vitamin composition of spent media from bacterial cultures grown in the presence of ^13^C-glucose or ^13^C-acetate and ^12^C vitamins. We analyzed the *m/z* traces of endogenous pantothenate (9 × ^13^C) as well as chimeric pantothenate (6 × ^13^C). We found that endogenous pantothenate concentration decreased when 100 μM ^12^C-pantothenate was present (Fig. 4*A*). The decrease was explained - as expected based on the pantothenate salvage mechanism we propose above—by the accumulation of partially labeled pantothenate in the medium (^13^C-pantoate-^12^C-β-alanine; Fig. S4*A*).

The labeling approach also allowed us to study whether other endogenous vitamins were secreted to the medium in the presence of exogenous vitamin sources and hence to address whether endogenous vitamin synthesis was downregulated. To this end, we analyzed spent culture media for endogenously produced (and hence U^13^C labeled) vitamin precursors in bacterial cultures grown in the presence of supplemented vitamins (U^12^C labeled). We found biotin, niacin, and riboflavin in the spent media, indicating that they were secreted and thus likely overproduced in the cells. Downregulation of a biosynthetic pathway for a vitamin would then lead to lower vitamin pools in the culture media. This pattern was observed for biotin supplementation: When 1 μM of ^12^C-biotin was supplemented, ^13^C-biotin accumulated in the medium, and 10 and 100 μM of biotin supplementation led to decreased secreted biotin (Fig. 4*B*). For *E. coli* cells grown on acetate, niacin secretion also followed this pattern, but endogenous niacin accumulated in the medium when cells were grown on glucose (Fig. 4*B*). Our findings indicate that overflow of biotin and niacin (on acetate) was downregulated by vitamin supplements, which is consistent with the literature on the regulation of these two pathways (5, 36). Finally, supplemented riboflavin, which was not observed to be taken up or incorporated into FAD or FMN, did not affect riboflavin overflow (Fig. 4*B*).

Our results indicate that although most vitamin production was reduced in the presence of supplements, they were still secreted into the media. To investigate whether vitamin supplementation suppresses the biosynthesis of vitamin precursors, we revisited the intracellular metabolomics data presented in Figure 1*B* to identify peaks corresponding to the expected *m/z* values of all precursors on the vitamin biosynthesis pathways (37, 38). We putatively identified 16 unique precursor metabolites for vitamins (Fig. S5). The data indicate that these were robust to vitamin supplementation with the only statistically significant exception of (R)-2,3-dihydroxy-isovalerate) when cells were grown on acetate (Fig. S5). Vitamin supplementation may lead to reduced expression of genes for enzymes of the respective biosynthetic pathway, as recently described for thiamine (39). To address whether enzymes for coenzyme biosynthesis investigated here remain to be produced upon vitamin supplementation, we used publicly available proteomics data (40). Comparing cells grown without vitamins to cells grown with vitamins, we observed that enzymes catalyzing vitamin biosynthesis were present in both conditions (Fig. S6), indicating that they are not fully repressed in the presence of vitamins. These orthogonal data are consistent with our observation that the majority of vitamin precursors are present in cells supplemented with vitamins, although in some cases downregulated (Figs. 3 and S5).

### Vitamin incorporation into coenzymes in diverse bacteria

**Figure 5:**
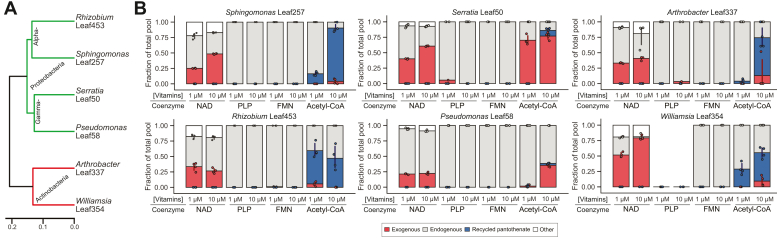
**Vitamin incorporation in prototrophic leaf isolates.***A*, phylogenetic tree of isolates included in this study. *B*, coenzyme origin in environmental strains. Strains were cultivated in a minimal medium supplied with ^13^C glucose as a carbon source. Vitamins (^12^C) were supplemented at either 1 or 10 μM. Data shown are from liquid cultures at the mid-exponential growth phase. For each bar in the stacked bar plots, samples with detectable levels of each coenzyme isotopologue are shown out of n = 4.

Our finding that supplemented pyridoxal, niacin, and β-alanine moiety of pantothenate are incorporated into their respective coenzymes in *E. coli* provokes the question of whether our observations regarding coenzyme homeostasis also expand to other bacteria. We addressed this question using six prototrophic bacteria that were isolated from *Arabidopsis thaliana* leaf surfaces and belong to different phylogenetic groups (27, 41). We cultivated these using U^13^C-glucose as a carbon source, adding either 1 μM or 10 μM U^12^C-vitamins as above for *E. coli.* Most strains incorporated niacin into NAD(H), but not exclusively as was observed in *E. coli* (Fig. 5). When vitamin concentration was increased from 1 to 10 μM, most species produced 10 to 20% more of their intracellular coenzyme from the externally supplied vitamins. None of the strains studied here incorporated pyridoxal or riboflavin (Fig. 5). For acetyl-CoA, we observed that Gammaproteobacteria (*Serratia* Leaf50 and *Pseudomonas* Leaf58) predominantly incorporated the intact pantothenate supplement, whereas CoA was built from β-alanine originally derived from pantothenate in Alphaproteobacteria and Actinobacteria (Fig. 5), indicating pantothenate cleavage and reconstitution is widespread beyond the observations initially made for *E. coli* in this study.

## Discussion

Vitamin supplements are often added to bacterial cultures. Thus far, quantitative data describing how the addition of vitamins affects cellular metabolism, how the requirements relate to *in vitro* observed K_m_ values of related enzymes and transporters, or to which extent they are sensed and incorporated by bacteria. For example, biotin and niacin are known to alter gene expression at both the transcriptional and translational levels, implying that cells are able to sense added vitamins in their environment (39, 42, 43, 44, 45). Here, we studied coenzyme pool sizes in different environmental conditions and monitored uptake and secretion of coenzyme precursors using a metabolic tracer approach.

We found that most coenzyme pools are stable when comparing different carbon sources and growth rates (Fig. 1*A*). This finding is congruent with recent data showing that the concentration of few metabolites depends on cellular physiology (19; see our dedicated analysis on coenzymes using these data in Fig. S7) as opposed to the proteome where many proteins correlate with growth rate or the carbon source (40). Although coenzyme pool sizes were similar during growth on different carbon sources and vitamin supplementation schemes, the observed two-fold increase in the NAD pool may be biologically meaningful. Biological effects depend on the concentration and the K_m_ values of the corresponding enzymes as was assessed globally in *E. coli* (18, 46). Seven enzymes in one or both of the tested conditions are predicted to become saturated upon increased coenzyme pool, for example, essential reactions such as 1-pyrroline-5-carboxylate reductase, glutamate synthase, glucose 6 phosphate dehydrogenase, and THF oxidoreductase (32, 47). Some of them, such as THF oxidoreductase and 6-phosphoglucose dehydrogenase may utilize both NAD(H) and NADP(H) (48). Change in concentration from sub-saturating to saturating of enzymes may lead to an increase in flux through that reaction (18). Exerting flux control *via* coenzymes could be beneficial for bacteria when a systems-level (as opposed to individual reactions) control of fluxes is required as each coenzyme affects many reactions. Therefore, fluctuations in coenzyme abundance can lead to drastic changes in the operation of the metabolic network. We observed generally lower coenzyme pools on glycerol and xylose-grown cells when compared to glucose-grown cells, indicating that enzyme saturation may be lower during growth on glycerol or xylose, allowing for more rapid changes in flux directionality or reaction rate (Fig. 1*A*).

Our findings indicate that coenzyme metabolism is modulated as a combined effect of carbon source, growth rate, and vitamin concentration. We found that the fraction of total coenzyme pools produced from vitamins increases with elevated extracellular vitamin concentration (Figs. 3 and 5). The metabolic tracer approach allowed us to determine the supplementation level at which the detected intracellular coenzyme pool was entirely derived from supplemented precursors. We included both glucose and acetate as carbon sources to modulate the growth rate and concomitantly the coenzyme synthesis rate of *E. coli* by a factor of 2 (2, 49), which we rationalized could affect the incorporation pattern of supplemented vitamins into coenzymes. Vitamin incorporation profiles of niacin, pyridoxal, and riboflavin in cells grown on acetate were similar to glucose, and therefore the growth rate does not generally affect vitamin incorporation (Figs. S1 and 2D). Intracellular NAD and PLP pools were fully derived from vitamins when niacin and pyridoxal were supplemented at 10 μM. Riboflavin was not incorporated into FMN or FAD (Figs. 3 and S2), consistent with previous studies that identified no riboflavin transporters in *E. coli* (50).

As opposed to other vitamin–coenzyme pairs where the vitamin was either incorporated or not, we found that *E. coli* as well as other bacteria preferentially use the β-alanine moiety of the external pantothenate molecule to build CoA instead of incorporating the fully formed pantothenate supplement. The unexpected labeling pattern of CoA was more pronounced when we grew the cells on ^13^C-labeled acetate as the carbon source (Fig. 2*D*): For CoA to be entirely derived from extracellular pantothenate-derived β-alanine, 10 μM of the vitamin was required when cells were grown on acetate and 100 μM when they were cultivated on glucose. There is a known enzyme activity in *Pseudomonas fluorescens* that degrades pantothenate, pantothenase (51, 52). The identity of the protein is currently unknown. Pantothenate kinase, the first reaction from pantothenate to CoA, is the rate-limiting step in CoA biosynthesis and is regulated by CoA (6). Highly active pantothenate synthesis combined with tightly regulated downstream reaction can explain why intracellular pantothenate is continuously degraded, only to be reconstituted with endogenous pantoate, of which a 15-fold excess is synthesized (6). We observed more pantothenate recycling in cells grown on acetate than in cells grown on glucose. While the potential metabolic mechanism of this physiology-dependent difference in pantothenate recycling remains elusive, it might be due to lower biosynthesis rates and therefore more strictly regulated pantothenate kinase activity in these cells (2), or due to physiological differences such as decreased precursor availability.

Prokaryotes as well as eukaryotes are known to salvage biosynthetic intermediates (53, 54, 55, 56, 57, 58). In the coenzyme context, salvage pathways refer to a series of dedicated reactions that reconstitute a partially degraded coenzyme. Several such pathways are known for coenzymes. NAD salvage pathway degrades NAD to 1-(β-D ribofuranosyl)nicotinamide, which is then rebuilt to NAD, and CoA salvage proceeds from 4′-phosphopantetheine (59). Of coenzymes not studied here, cobalamin has a salvage pathway that begins with a stable cobamide, which is converted to a *de novo* intermediate, Coβ-5′-deoxyadenosylcobyric acid, in a series of dedicated salvage reactions (60). Recent studies found that the equilibrium between B12 producers, salvagers, and auxotrophs is important for the stability of microbial communities (61, 62). Our observation of pantothenate reassembly with exogenous β-alanine derived from supplemented pantothenate and endogenous pantoate is unique in so far that it presents a salvage mechanism that centers around the vitamin precursor instead of the fully-formed coenzyme.

Metabolites are secreted from growing cells and are present in the supernatants of bacterial cultures (63). Metabolic imbalances lead to an intracellular accumulation of metabolites, which is then mitigated by excretion. For example, pantoate overflow has been shown to lead to pantothenate secretion in quantities sufficient in providing mammalian hosts with the vitamin (6). The approach applied here allowed us to discriminate between endogenous and supplemented vitamins in culture supernatants. We found vitamin secretion even at 100 μM extracellular vitamin concentrations, ruling out passive transport and suggesting that vitamins are actively exported from cells. We also analyzed the expression levels of vitamin biosynthetic proteins in published data as well as precursors to vitamins in the data acquired in this study (Figs. S5 and S6), both datasets support that vitamin biosynthesis remain active in the presence of vitamin supplements. Moreover, we detected riboflavin secreted to the medium, although it was not taken up. Indeed, a riboflavin exporter, YeeO, has been described in *E. coli* (64). Therefore, our results indicate that vitamin secretion systems are energy-coupled and orthogonal to the vitamin uptake systems.

In addition to *E. coli* K-12, we tested diverse environmental strains and found that they incorporated fewer vitamins, both quantitatively and qualitatively. The data presented here indicate that riboflavin and pyridoxal are generally not incorporated (Fig. 5*B*). This is in line with our previous observation that riboflavin accumulates in bacterial co-cultures, and may explain the relatively narrow range of vitamin auxotrophy observed in the tested environmental strains (27, 65). Pyridoxal transporters, although present in *E. coli*, were likely lacking in all other species included in this study under the experimental conditions tested, as no pyridoxal was incorporated into PLP (Figure 3, Figure 4, Figure 5). Pantothenate reconstitution shunt described here for *E. coli* was also observed in four out of the six tested species, indicating that the mechanism is widespread. Whether there is an underlying metabolic mechanism unifying bacteria that recycle pantothenate remains elusive.

Coenzyme pools inside *E. coli* and other microorganisms are maintained at surplus, likely to ensure the robustness of coenzyme levels upon cell division (2, 27). Our data indicate that there is an additional mechanism to ensure coenzyme levels, which is the overproduction of dedicated precursors, offering a reservoir for rapid synthesis into functional coenzymes when necessary. This mechanism may contribute to the widespread occurrence of auxotrophy in bacteria associated with bacterial communities: organisms that produce vitamins will also concomitantly secrete them, leading to a vitamin supply in the environment.

## Experimental procedures

### Microbial culture

All strains were cultivated in media buffered with phosphates (2.4 g/l K_2_HPO_4_, 2.08 g/l NaH_2_PO_4_·2H_2_O) with mineral salts (1.62 g/l NH_4_Cl, 0.2 g/l MgSO_4_·7H_2_0). Glucose was added at 20 mM and acetate at 60 mM. For all media components, 10× stocks were prepared, dissolved in ddH_2_O, and filter sterilized. The vitamin concentrations in the “1 μM vitamins” condition were: D-pantothenic acid hemi calcium salt 1.05 μM, biotin 0.41 μM, riboflavin 1.06 μM, thiamine **·** HCl 1.19 μM, pyridoxal **·** HCl 0.98 μM, p-amino benzoic acid 1.09 μM, cobalamin 0.14 μM, lipoic acid 0.24 μM, niacin 1.22 μM, folic acid 0.23 μM. For riboflavin, 100× stocks were prepared. All stock solutions were filter sterilized. For all other vitamins, 1000× stocks were prepared. Trace elements were added in following final concentrations: 15.65 μM, ZnSO_4_ **·** 7 H_2_O [VWR International], 12.61 μM CoCl_2_ **·** 6 H_2_O, 5.09 μM MnCl_2_, 16.17 μM H_3_BO_3_, 1.65 μM Na_2_MoO_4_ **·** 2 H_2_O [Fluka], 1.20 μM CuSO_4_ **·** 5 H_2_O, 20.41 μM CaCl_2_ **·** 2 H_2_O. Iron compounds was added in the following final concentrations: 40.30 μM FeSO_4_ **·** 7 H_2_O, 40.30 μM Na_2_EDTA **·** 2 H_2_O.

U^13^C-glucose and U^13^C-acetate were purchased from EURISO-TOP GmbH. Unless stated otherwise, other chemicals such as vitamins and buffer salts were obtained from Sigma-Aldrich. *E. coli* strain BW25113 was used throughout the study (47). Natural isolates were obtained from At-LSPHERE culture collection (41).

### Growth rate assays

All growth assays were performed at 28 °C for At-LSPHERE strains and 37 °C for *E. coli*. The turbidity of shake flask cultures was determined by measuring the optical density at 595 nm (=”OD_600_”) in semi-micro cuvettes (Bio-Greiner) using a Biophotometer Plus (Eppendorf). Samples were diluted as appropriate to keep the OD readings in the linear range. To analyze the data, linear regression on ln transformed data was carried out using linear_regression function from sklearn. Data points that lay outside of the log-linear scale were omitted. Growth rates were only calculated for cultures that performed at least two doublings in the exponential phase.

### Metabolome sampling

Quantitative analyses were performed by extracting exponentially growing cells in liquid cultures *via* a fast filtration protocol. Around 250 μg of cell material (corresponding to 1 ml of OD1) was transferred to a regenerated cellulose filter, which was pre-washed with 50 °C ddH_2_O. Cells were then washed with 10 ml of ddH_2_O pre-warmed to the temperature of the culture (37 °C for *E. coli*, 28 °C for At-LSPHERE strains). Overall, the cells spend <10 s on filter. The filter was then transferred to 8 ml of quenching and extraction solution (60:20:20 MeCN:MeOH:0.5 M formic acid (66)) that was pre-cooled to −20 °C and transferred onto ice minutes before sampling. For samples dedicated to pool size determination, metabolite extract from 125 μg of 13C-labeled *E. coli* was added as an internal standard. These extracts were prepared by the same protocol as here for *E. coli* for >15 generations on U^13^C D-glucose, and stored in −80 °C. The sample was left to extract on ice for 10 min until the filters were removed. The solutions were lyophilized at −80 °C overnight, re-dissolved in 250 μl of ddH_2_O, and stored in −80 °C until LC/MS analysis.

For exometabolome analysis, supernatants were sampled simultaneously to intracellular metabolites. To this end, the same culture volume that was transferred to a filter, was transferred to a 2.0 ml Eppendorf tube. After a brief centrifugation (4 °C, 10′000 rpm, 5 min), the supernatant was transferred to a clean 2.0 ml Eppendorf tube. The liquid was evaporated using a SpeedVac system (Eppendorf) and resuspended in 150 uL ddH_2_O. Supernatant samples were stored at −20 °C until analysis.

### LC/MS analysis

All targeted measurements for coenzymes, vitamins, and vitamin precursors originating from vitamin-supplemented samples were conducted using UHPLC-HRMS. For each sample, 5 μl of metabolite extract was separated in liquid chromatography achieved with a Thermo Ultimate 3000 UHPLC system (Thermo Scientific) at a flow rate of 500 μl min^−1^. The C18 reversed phase (C18RP) separation was achieved using a Kinetex XB-C18 column (particle size 1.7 μm, pore size 100 Å; dimensions 50 × 2.1 mm, Phenomenex) as described before (67). Mobile phase A was 50 mM formic acid at pH 8.1 (ammonium hydroxide), and mobile phase B was methanol. The following gradient was applied: from 0 to 2.1 min, 0% B. B was then increased to 5% at 2.2 min, 23% at 6.6 min, and 80% at 11 min. B was maintained at 80% until 11.9 min and brought to 0% at 12 min. The column was then equilibrated for 2.1 min. The length of the method was 14.1 min. For mass analysis, LC instrument was coupled to a Thermo QExactive Plus instrument (Thermo Fisher Scientific), and the mass spectrometer was operated in both positive and negative mode at mass resolution of 35,000. Heated electro-spray ionization probe was used applying the following source parameters: vaporizer 350 °C; aux gas 20; ion spray voltage +3.00 kV, sheath gas, 50; sweep gas, 0; radio frequency level, 50.0; capillary temperature, 275 °C.

To determine pool sizes in samples grown on different carbon sources (Fig. 1*A*), nanoscale high-performance liquid chromatography–high-resolution mass spectrometry (Nanoscale HPLC-HRMS) was achieved by liquid chromatography EASY-nLC 1000 (Thermo Scientific) system connected to a Q Exactive Plus (Thermo Scientific) instrument. Metabolite separation was carried out on a C18 column (Dr Maisch Reprosil-Gold 120, 1.9 μm, 50 × 2 mm, Morvay Analytik) as described previously (68). Briefly, the method relies on ion pairing, which was achieved using solvent A (1.7 mM TBA): TBA was dissolved in 1.5 mM aqueous acetic acid, and pH was adjusted to 9.0 with ammonium hydroxide. Metabolites were eluted using methanol as solvent B on the following multistep gradient: 0 min, 3%; 27 min, 40%; 40 min, 90%; 45 min, 90%; 46 min, 3%; 56.5 min, 3%. The sample injection volume was 5 μl and the flow rate was 400 nl min^−1^. The mass acquisition was operated in negative Fourier transform mass spectrometry in full MS scan mode.

Targeted tandem MS measurements were performed by PRM (parent reaction monitoring) using the UHPLC-HRMS. All positionally labeled products were monitored at once in data-independent acquisition by setting the isolation window to 30 *m/z*. In negative mode, a stepped normalized collision energy was applied (nce = 25, 30, 35). Compounds were preferentially analyzed in positive mode; however, NADP and PLP could only be detected in negative mode. In positive mode, parent ions were isolated and fragmented using parameters in Table S2.

### LC/MS data analysis

Retention times of coenzymes and vitamins were determined using analytical standards. Peak areas were determined *via* linear integration as described in the emzed documentation (68) (https://emzed.ethz.ch/emzed_tutorial/). For vitamin measurements, baseline correction was applied due to noise in the chromatogram. For fragmentation analysis, relevant fragments were identified using published spectra in Metlin database (69). For compound-specific measurement parameters, see Table S2.

To determine relative pool sizes, we took advantage of the isotope dilution method (29). As the metabolite samples were spiked with U^13^C-labeled *E. coli* extract as an internal standard, we calculated the ratio between the peak area originating from the internal standard and the naturally labeled peak originating from the sample and multiplied by 2 (50% less internal standard was added when compared to the amount of sample). Fractional labeling was inferred from peak areas of compounds for *M* + 1.003355, *M* + 2∗1.003355, …, *M* + *N*∗1.003355 where *N* is the number of carbon atoms in the compound and *M* is the exact mass of a compound minus the mass of a proton (1.0072). Each area was divided by the total sum of all *N* isotopologues to define the percentage of the isotopic cluster originating from each isotopologue.

### Analysis software and statistical analysis

Unless otherwise stated, all analyses were performed on a Windows machine running Python 3.8 *via* Anaconda3 using custom scripts. Data were handled in pandas dataframes (V 1.1.3), for numerical computing numpy library (V 1.20.1) was used, and linear regression and multiple testing correction were performed *via* the sklearn and statsmodels (V 0.23.0 and V 0.12.0, respectively) implementations. For statistical testing, scipy (V 1.5.2) implementations were used. APIs were queried *via* requests (V 2.22.0), and KEGG *via* Biopython (V 1.76). BRENDA database was queried *via* SOAP access (https://www.brenda-enzymes.org/soap.php).

## Data availability

The mass spectrometry metabolomics data are deposited in MetaboLights (https://www.ebi.ac.uk/metabolights/index) with the dataset identifier MTBLS8121. Upon request, additional data and material will be made available.

## Supporting information

This article contains supporting information (15, 19, 42).

## Conflict of interest

The authors declare no competing interests.
