# Supplementary material for: Coenzyme biosynthesis in response to precursor availability reveals incorporation of β-alanine from pantothenate in prototrophic bacteria
Source: J Biol Chem. 2023 Jun 12;299(8):104919. doi: 10.1016/j.jbc.2023.104919 (PMC10393543; doi:10.1016/j.jbc.2023.104919)
Supplement: Supporting Figures S1–S7 and Tables S1 and S2 [file mmc1.pdf]

Supporting information

Coenzyme biosynthesis in response to precursor availability reveals incorporation of β-alanine from pantothenate in prototrophic bacteria

Birgitta Ryback[1,2] & Julia A. Vorholt[1]*

[1] Institute of Microbiology, ETH Zurich, 8093 Zurich, Switzerland

[2] Current address: Department of Cancer Biology, Dana-Farber Cancer Institute, Boston, MA, USA

*For correspondence: Julia A. Vorholt, jvorholt@ethz.ch

Supplementary Tables

Supplementary Table 1 Enzymes that become saturated with coenzymes due to increased NAD pool size. Absolute concentrations without vitamin supplements are taken from Bennet et al. (15).

EC	Coenzyme	Concentration [M] (15)	K_m [M]	Condition	Description
1.1.1.49	NAD+	2.43E-3	5.09E-3	Acetate	6-phosphoglucose dehydrogenase G6PDH (Zwf)
1.5.1.2	NADH	1.34E-4	1.83E-4	Acetate	1-pyrroline-5-carboxylate reductase (ProC)
1.5.1.3	NADH	1.34E-4	2.94E-4	Acetate	5,6,7,8-tetrahydrofolate: NADP+ oxidoreductase (THF oxidoreductase) (FolA)
1.6.5.2	NADH	1.34E-4	1.5E-4	Acetate	1,4-benzoquinone reductase (WrbA)
1.1.1.17	NADH	8.32E-05	9.90E-05	Glucose	mannitol-1-phosphate dehydrogenase (MtlD)
1.1.1.49	NAD+	2.55E-3	5.09E-3	Glucose	6-phosphoglucose dehydrogenase (Zwf)
1.1.1.58	NADH	8.32E-05	8.50E-05	Glucose	D-tagaturonate reductase (UxaB)
1.4.1.13	NADH	8.32E-05	1.02E-4	Glucose	glutamate synthase (GltB)
1.5.1.2	NADH	8.32E-05	1.83E-4	Glucose	1-pyrroline-5-carboxylate reductase (ProC)
1.5.1.3	NADH	8.32E-05	2.94E-4	Glucose	5,6,7,8-tetrahydrofolate: NADP+ oxidoreductase (FolA)
1.6.5.2	NADH	8.32E-05	1.5E-4	Glucose	1,4-benzoquinone reductase (WrbA)

Supplementary Table 2 MS^2 parameters for coenzymes. The instrument was operated in positive mode (for details see Material and Methods).

m/z	Start [min]	End [min]	N(CE)	Compound name
821.66379	1.8	2.25	30	Acetyl-CoA
777.89732	2.4	3	30	CoA
799.7097	4	5.2	20	FAD
465.64032	3.7	4.1	20	FMN
674.64434	0.5	1.2	25	NAD
880.17876	0.5	1.2	30	Succinyl-CoA

Supplementary Figures

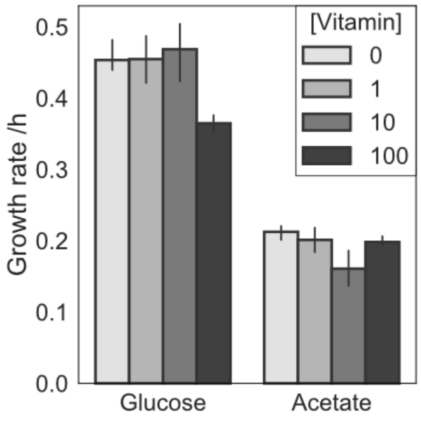

Supplementary Fig. 1 Growth rate of *E. coli* when grown on different carbon sources and vitamin concentrations. The vitamins represent a mixture of pantothenic acid, biotin, riboflavin, thiamine, p-amino benzoic acid, cobalamin, lipoic acid, niacin and folic acid (see Experimental procedures for details). Vitamin concentrations in the legend are given in micromoles/liter.

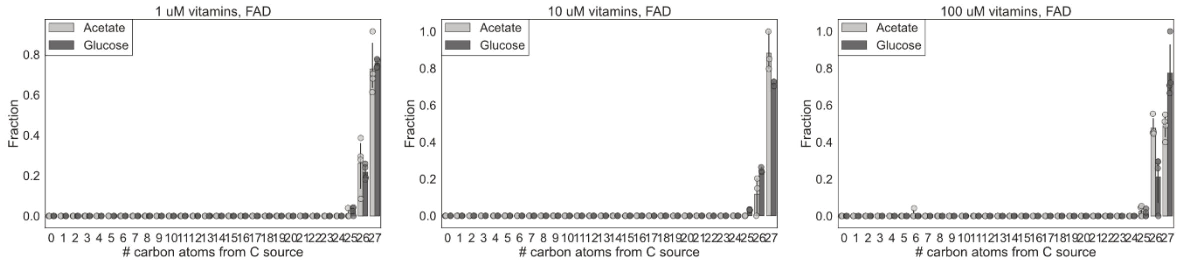

Supplementary Fig. 2 Riboflavin is not incorporated into FAD. Naturally labelled (99% ^{12}C) riboflavin (number of carbon atoms: 17) was supplemented cultures grown in ^{13}C labelled carbon sources. If riboflavin was incorporated, the resulting FAD isotopologue would therefore be 10 labelled carbons. Instead, fully ^{13}C labelled FAD was detected, indicating no riboflavin incorporation.

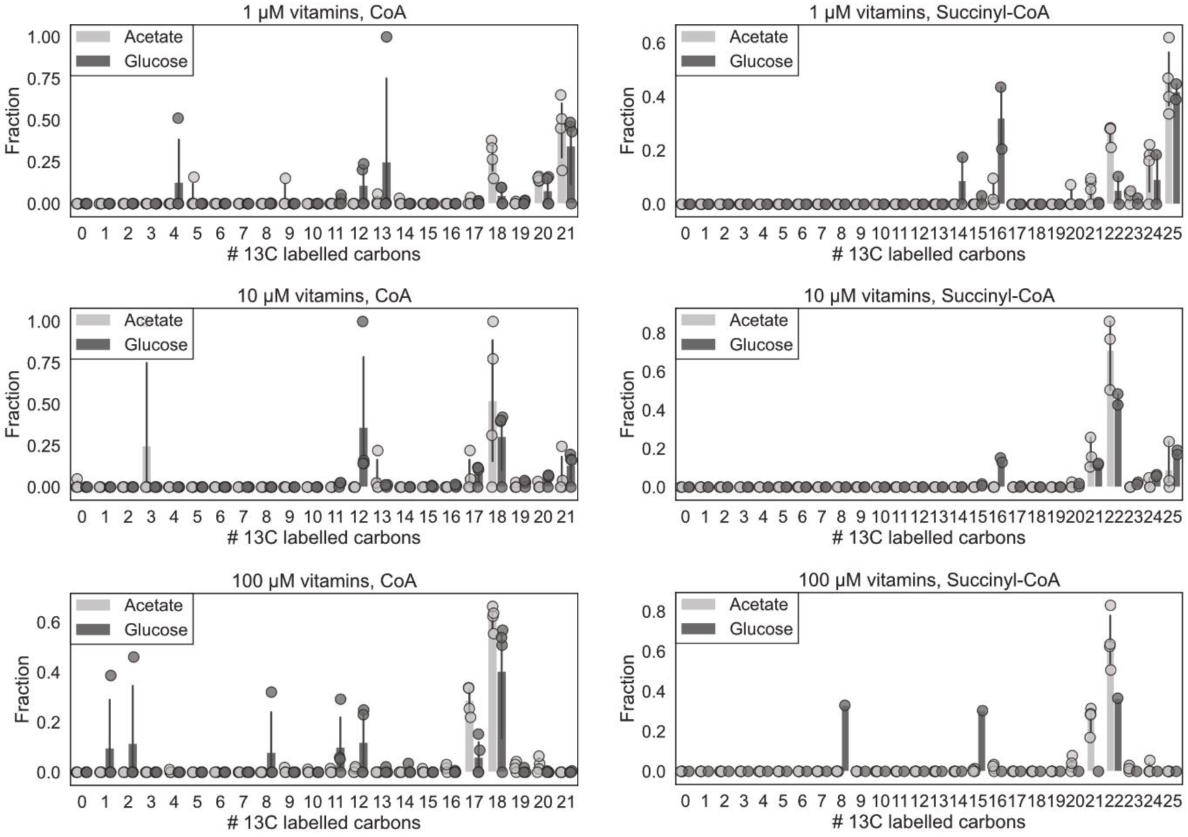

Supplementary Fig. 3 Pantothenate is degraded and β-alanine is incorporated into CoA thioesters. Naturally labelled (99% ^{12}C) pantothenate (9C) was supplemented cultures grown in ^{13}C labelled carbon sources. If pantothenate was incorporated, the resulting R-CoA isotopologue would therefore be labelled with N-9 labelled carbons where N is the number of carbon atoms in R-CoA. Instead, fully N-3 labelled CoA esters were detected. Fractional labelling patterns of free CoA and succinyl-CoA.

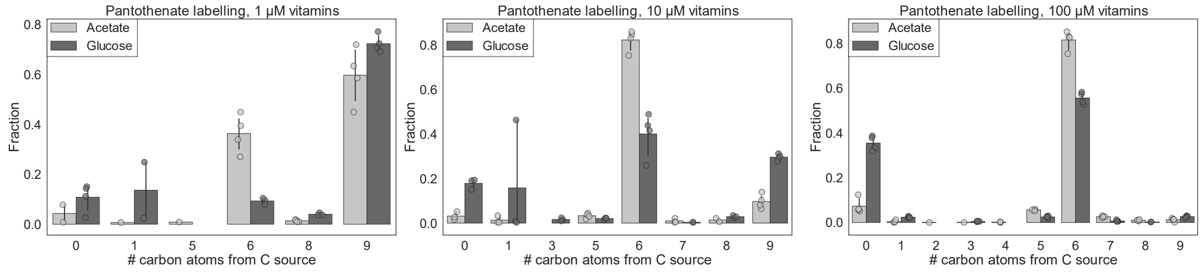

Supplementary Fig. 4 Fractional labelling of intracellular pantothenate in increasing vitamin supplementation. Cells were supplemented with ^{12}C labelled vitamins and ^{13}C labelled carbon sources. Therefore, supplemented pantothenate is visible at 0 labelled fraction and endogenously synthesized pantothenate at 9 labelled fraction. However, when pantothenate is supplemented, most intracellular pantothenate is labelled with 6 heavy carbons, indicating incorporation of 3 carbons from the supplement.

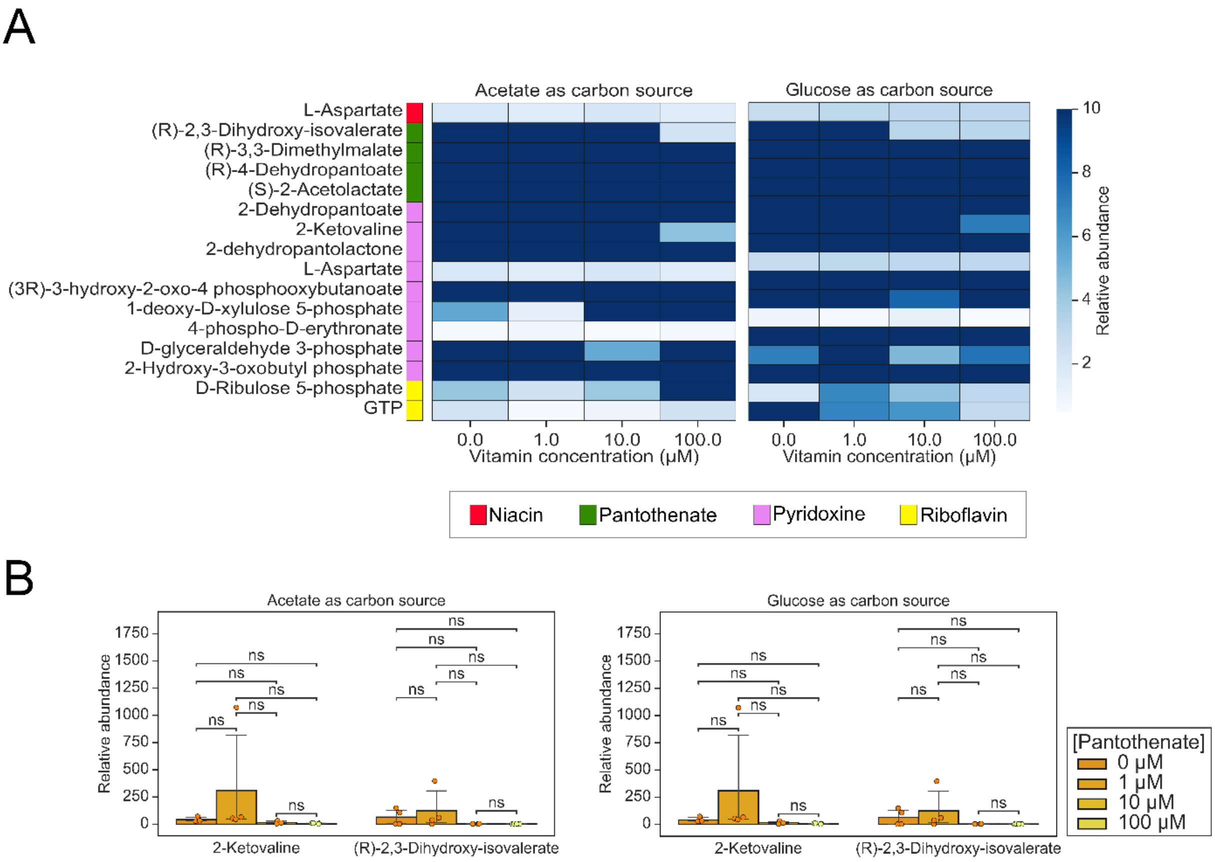

Supplementary Fig. 5 Vitamin precursor analysis in cells grown with vitamin supplements. A, heatmap summarizing four experiments for each of the observed vitamin precursors. A downward trend in the relative abundance of a metabolite may indicate a regulation point. Such trend was only found for two of these precursors, 2-ketovaline and (R)-2,3-dihydroxy-isovalerate. B, Statistical comparison of 2-ketovaline and (R)-2,3-dihydroxy-isovalerate pool sizes in cultures supplemented with vitamins.

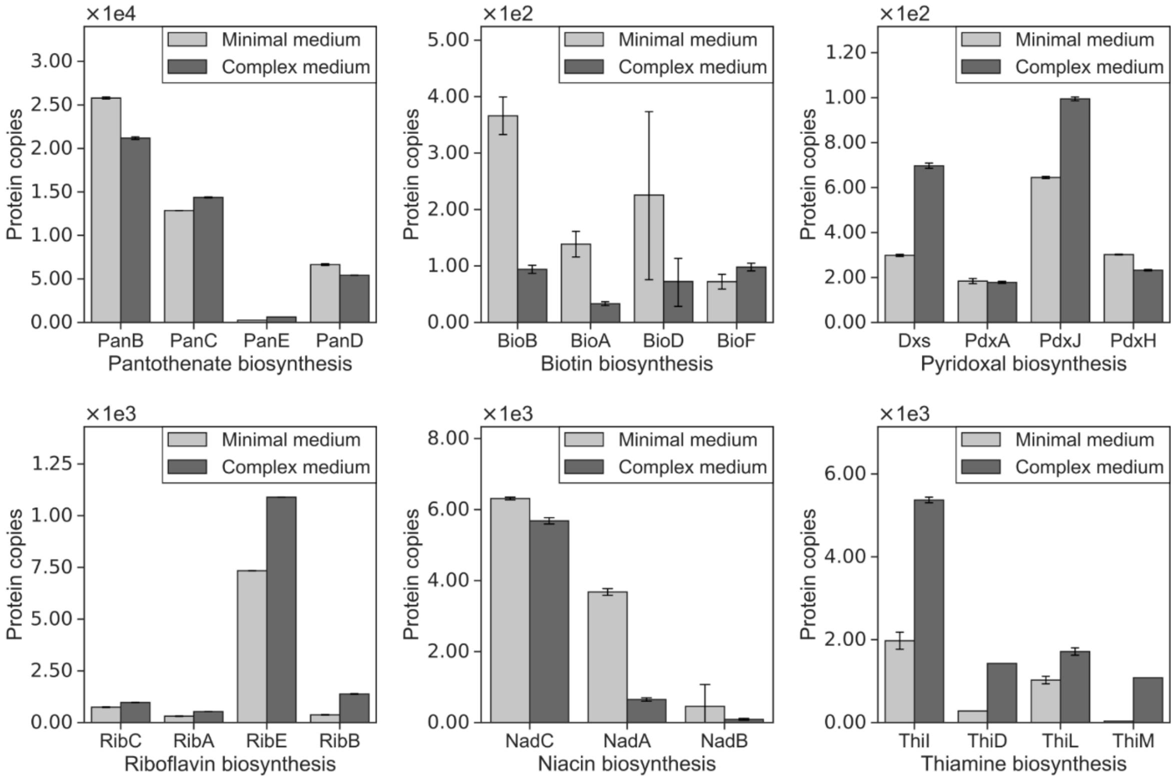

Supplementary Fig. 6 Copy numbers of enzymes involved in biosynthesis of vitamins indicated on the *x* axis. Data from ref. 42. Minimal medium refers to M9 medium without vitamins (with glucose as the carbon source) and complex medium to LB which contains vitamins.

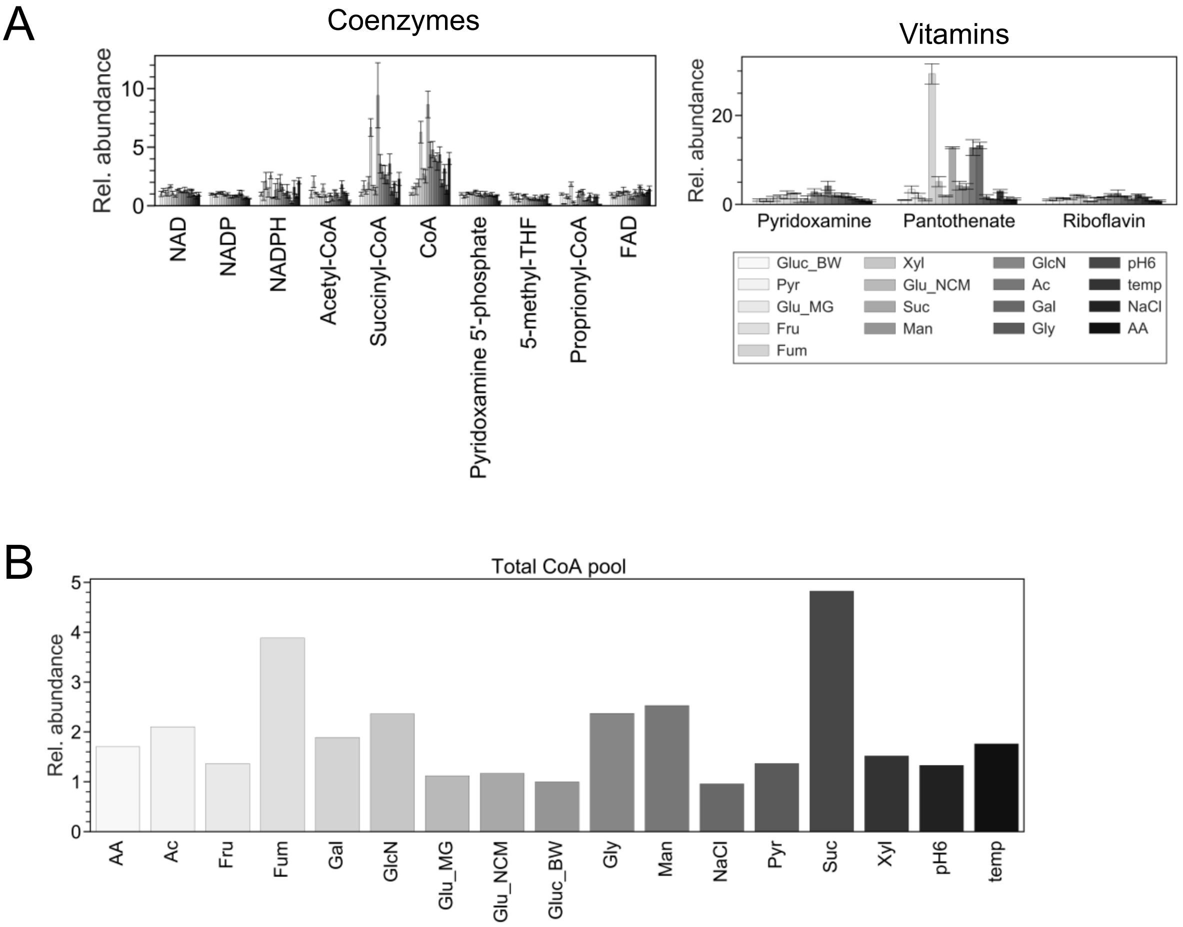

Supplementary Fig. 7 Relative coenzyme concentrations. Source data from conditions of exponentially growing cells are taken from ref. 19. **a)** Relative abundances of coenzymes and vitamins compared to glucose-grown cells in *E. coli* BW2113. Conditions in the legend include different carbon sources and stressors. In the glucose condition, three different model strains were measured (labelled as Gluc_X). The last column of the legend is dedicated to metabolite pools in *E. coli* grown in the presence of stressors. Stressors used were pH 6 (slightly acidic pH), temp (high temperature, 42°C), NaCl (sodium chloride stress). Last condition refers to glycerol medium with amino acids. **b)** Total CoA pool (sum of acetyl-CoA, succinyl-CoA, CoA, and propionyl-CoA) in different conditions.
